# Mechanisms of endothelial dysfunction in rheumatoid arthritis: lessons from animal studies

**DOI:** 10.1186/ar4450

**Published:** 2014-01-24

**Authors:** Perle Totoson, Katy Maguin-Gaté, Clément Prati, Daniel Wendling, Céline Demougeot

**Affiliations:** 1 EA 4267 « Fonctions et Dysfonctions Epithéliales », UFR Sciences Médicales et Pharmaceutiques, 19 rue Ambroise Paré, bâtiment S, 25030, BESANCON cedex, FRANCE; 2 Service de Rhumatologie, CHU Minjoz, 3 Boulevard Alexandre Fleming, 25030, BESANCON, France

## Abstract

Rheumatoid arthritis (RA) is a chronic systemic inflammatory disease characterized by articular and extra-articular manifestations involving cardiovascular diseases (CVDs), which account for 30% to 50% of all deaths. In patients with RA, atherosclerosis lesions occur earlier and have a more rapid evolution than in the general population. Beyond mortality, the impact of CVD on quality of life, combined with the associated increase in health-care costs, renders CVD in RA a major public health problem. Recent studies showed that patients with RA are characterized by the presence of endothelial dysfunction (ED), which is recognized as a key event in the development of atherosclerosis. By definition, ED is a functional and reversible alteration of endothelial cells, leading to a shift of the actions of the endothelium toward reduced vasodilation, proinflammatory state and proliferative and prothrombotic properties. Although the improvement of endothelial function is becoming an important element of the global management of patients with RA, the mechanistic determinants of ED in RA are still poorly understood. Animal models of RA provide the unique opportunity to unravel the pathophysiological features of ED in RA. The present review summarizes the available data on mechanisms underlying ED in animal models of RA and proposes attractive prospects in order to discover novel therapeutic strategies of RA-associated ED.

## Introduction

Rheumatoid arthritis (RA) is a chronic systemic inflammatory disease characterized by articular and extra-articular manifestations, including cardiovascular diseases (CVDs), which account for 30% to 50% of all deaths [1]. Recent studies showed that atherosclerosis lesions occur earlier and have a more rapid evolution in patients with RA than in the general population [1]. Of interest, it is now established that RA is equivalent to type 2 diabetes as an independent risk factor for CVD [2,3]. Although widely investigated, the underlying causes of the increased prevalence of CVD among patients with RA remain to be elucidated. Nonetheless, despite changes in the course of the disease in recent years and new therapeutic options, there is still no evidence that any particular intervention can reduce CVD risk in RA [4]. Endothelial dysfunction (ED) is thought to be a key event in the development of atherosclerosis [5]. ED was first identified in patients with RA by Bergholm and colleagues in 2002 [6] and is now recognized as an important element of the cardiovascular (CV) risk in RA [7]. However, the precise pathophysiological mechanisms of ED in RA are still ill defined while their identification is a prerequisite for the discovery of drugs aiming to reduce CV risk in patients with RA. Because it is difficult to investigate these mechanisms in humans, studies on animal models of RA are useful for surrogate studies. The present review aimed to synthesize available data on ED and its potential mechanisms in animal models of RA.

## Animal models of arthritis used to study endothelial dysfunction

Most of the studies were performed in the rat model of adjuvant-induced arthritis (AIA) initially described by Pearson [8]. This model is induced by a single injection of a suspension containing heat-killed *Mycobacterium* emulsified in oil into the tail base or the hind-paw footpad. It is characterized by reliable, rapid onset and progression and easily measurable polyarticular inflammation, marked bone resorption, and periosteal bone proliferation [9]. Clinical signs of polyarthritis usually appear about 10 to 12 days after injection. The AIA model is T cell- and neutrophil-dependent and complement-independent [9]. Few experiments were conducted in the mouse model of collagen-induced arthritis (mCIA). In this model, genetically susceptible strains of mice are immunized with injection in the tail of heterologous type II collagen in complete Freund adjuvant, followed by a boost of collagen 3 weeks later [9]. Animals develop an autoimmune polyarthritis characterized by cartilage destruction, bone resorption, synovitis, and periosteal proliferation. Clinical signs of polyarthritis appear about 10 to 12 days after boosting [9]. The mCIA model involves T and B cells and is complement-dependent [9]. In the two models, the severity of arthritis is routinely assessed by determining the thickness of each limb and/or a clinical score or both, taking into account swelling and erythema of the four limbs. The clinical scores can be further divided in four grades from 0 (least severe) to 3 (most severe) [10].

## Endothelial dysfunction in animal models of arthritis

### Definition of endothelial dysfunction

**Figure 1:**
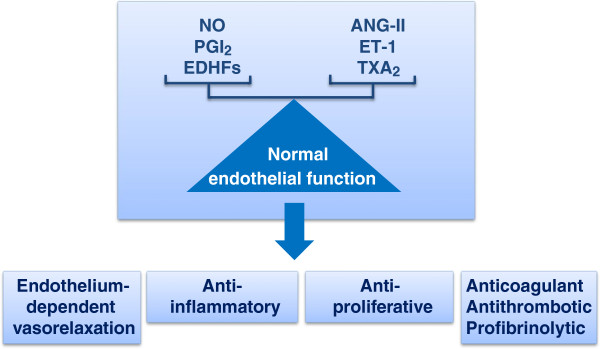
**Endothelium-derived factors and normal endothelial function.** ANG-II, angiotensin II; EDHF, endothelium-derived hyperpolarizing factor; ET-1, endothelin 1; NO, nitric oxide; PGI_2_, prostacyclin; TXA_2_, thromboxane A_2_.

The endothelium, once considered a mere selectively permeable barrier between bloodstream and vascular wall, is now recognized as a crucial homeostatic organ, fundamental for the regulation of the vascular tone and structure [11]. It senses mechanical stimuli, such as pressure and shear stress, and chemical stimuli, such as hormones and locally secreted vasoactive substances. In response to these stimuli, the endothelium releases factors that regulate vasomotor function, inflammatory processes, cell growth, and hemostasis (Figure 1). Among the vasodilator substances produced by the endothelium are nitric oxide (NO), prostacyclin (PGI_2_), and endothelium-derived hyperpolarizing factors (EDHFs). Vasoconstrictors include endothelin-1, angiotensin II (ANG-II), and thromboxane A_2_ (TXA_2_) [11]. ‘Endothelial dysfunction’ is a widely used term to describe any form of abnormal functional and reversible alteration of endothelial cells, leading to a shift of the actions of the endothelium toward reduced vasodilation, proinflammatory state, and proliferative and prothrombotic properties [12]. ED is an important early event in the pathogenesis of atherosclerosis, contributing to plaque initiation and progression [5]. In ‘traditional’ CVD, ED severity has a prognostic value for CV events and the correction of ED may be associated with reduced CV risk [13].

### Evidence of endothelial dysfunction in animal models of arthritis

Endothelial function was studied in the widely used model of isolated aortic rings that investigated the effects of constrictive or relaxant drugs in isometric conditions. The studies were conducted mainly on the AIA model, and the assessment of ED was made at a time at which inflammatory symptoms are maximal (that is, between day 24 and 35 after the injection of *Mycobacterium* suspension, approximately 14 to 21 days after the onset of arthritis). All studies unequivocally demonstrated a reduced acetylcholine (ACh)-induced endothelium-dependent vasorelaxation compared with control rats and therefore attested to the presence of ED at this stage of the disease (Table 1) [14-23]. It is noteworthy that the ED severity mirrors the arthritis severity. Indeed, at day 24 post-injection in AIA, no alteration of ACh-induced relaxation was found in grade 1 arthritic rats whereas rats with grade 2 or 3 arthritis exhibited a reduced relaxation to ACh [16]. Likewise, at day 35 post-injection, we identified an inverse correlation between the maximal effect of ACh and the arthritis grade [22]. Only two studies investigated ED in the model of mCIA. In one study conducted at a time corresponding to mild early-onset disease, ED was not observed [24] whereas in another study, aortic ED was identified 8 weeks after collagen injection [25].

**Table 1:** Studies on vascular reactivity in animal models of arthritis

**Authors (year)**	**Animal strain**	**Arthritis model *Arthritogenetic agent *** **( *injection zone *)**	**Time after induction of arthritis**	**Response to ACh**	**Response to SNP**	**Response to vasoconstrictors**
Fang *et al*. [14] (1991)	Male Wistar rat	AIA *M. butyricum* (*tail*)	Day 26	↓	=	PE = KCl =
Cinar *et al*. [15] (1998)	Male rat (strain NR)	AIA *M. tuberculosis* (*pad*)	Day 26	↓	=	PE ↑ KCl =
Ulker *et al*. [16] (2000)	Male Wistar rat	AIA *M. tuberculosis* (*pad*)	Day 29	↓ in grade 2 and 3 = in grade 1	↓ in grade 2 and 3 = in grade 1	PE ↑ in grade 2, PE ↓ in grade 3, KCl ↑ in grade 2, KCl = in grade 1 and 3
Can *et al*. [17] (2002)	Male Wistar rat	AIA *M. tuberculosis* (*pad*)	Day 26	↓	=	NR
Haruna *et al*. [18] (2006)	Male Lewis rat	AIA *M. butyricum* (*tail*)	Day 35	↓	=	NR
Haruna *et al*. [19] (2007)	Male Lewis rat	AIA *M. butyricum* (*tail*)	Day 35	↓	=	NR
Nozaki *et al*. [20] (2007)	Male Lewis rat	AIA *M. butyricum* (*tail*)	Day 24	↓	=	NR
Sakuta *et al*. [21] (2010)	Male Lewis rat	AIA *M. butyricum (tail*)	Day 35	↓	=	NR
Prati *et al*. [22] (2011)	Male Lewis rat	AIA *M. butyricum* (*tail*)	Day 35	↓	=	KCl =
Prati *et al*. [23] (2012)	Male Lewis rat	AIA *M. butyricum* (*tail*)	Day 35	↓	=	NE, ANG-II, ET-1=
Reynolds *et al*. [24] (2012)	Male DBA/1 mice	CIA *Chick collagen* (*NR*)	Day 24-27	=	=	Serotonin ↓, KCl ↓
He *et al*. [25] (2013)	Male DBA/1 mice	CIA *Bovine collagen (tail)*	Day 56	↓	=	NR

Vascular reactivity was studied in the model of isolated aortic rings. ↑, increased; ↓, decreased; =, unchanged; ACh, acetylcholine; AIA, adjuvant-induced arthritis; ANG-II, angiotensin II; CIA, collagen-induced arthritis; ET-1, endothelin-1; KCl, high potassium chloride; NE, norepinephrine; NR, not reported; PE, phenylephrine; SNP, sodium nitroprusside.

## Mechanisms of arthritis-induced endothelial dysfunction

### Decreased nitric oxide availability

**Figure 2:**
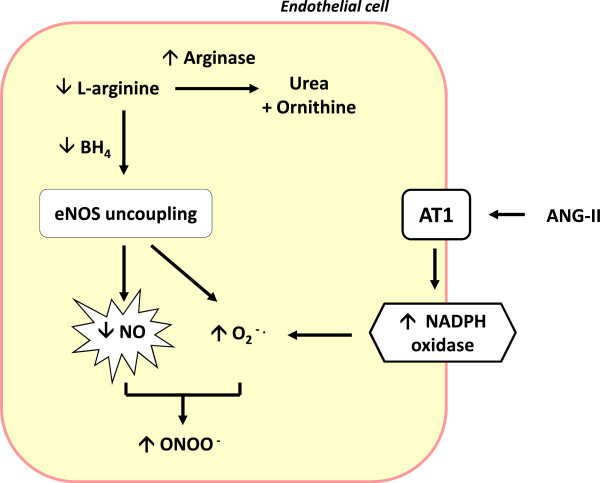
**Schematic representation of the mechanisms involved in decreased nitric oxide (NO) production in endothelial cells from arthritic rats.** Endothelial nitric oxide synthase (eNOS) catalyses the conversion of L-arginine to NO. The upregulation of arginase pathway and the deficit in the co-factor of eNOS tetrahydrobiopterin (BH_4_) cause uncoupling of eNOS to generate superoxide anions (O_2_^–^) which subsequently scavenge NO to generate peroxynitrite (ONOO^−^). Angiotensin-II (ANG-II) might amplify O_2_^–^ production by activating NADPH (reduced form of nicotinamide adenine dinucleotide phosphate) oxidase after ANG-II type 1 (AT1) receptor activation. Up and down arrows indicate increases or decreases in amount or activity (from [14-23]).

In large vessels such as the aorta, NO released by endothelial cells is a major contributor of vasorelaxation. The presence of ED in AIA rats suggests that the vascular bioavailability of NO is blunted (Figure 2). Production of NO by vessels can involve both endothelial NO synthase (eNOS) and inducible NOS (iNOS). The lack of effect of the selective iNOS inhibitor 1400 W on the ACh-induced vasodilation in AIA rats argues against a role of iNOS in AIA-associated ED [23]. Thus, decreased NO availability may result from different mechanisms, including decreased eNOS protein expression, decreased eNOS activity, decreased NO synthesis secondary to decreased availability of the NOS co-factor tetrahydrobiopterin (BH_4_), deficiency in intracellular L-arginine (the substrate of NOS), accumulation of the endogenous eNOS inhibitor asymmetric dimethylarginine, or inactivation of NO through excessive generation of superoxide anion (O_2_^–^) [26].

#### Endothelial nitric oxide synthase expression/activity

In aortas from AIA rats, eNOS expression was found to be unchanged [22] or increased [18,19] on day 35 post-injection. However, because eNOS is highly regulated at the post-transcriptional level, eNOS expression is not a good predictor of its activity [27,28]. It is therefore more interesting to measure the ratio between the serine 1177-phosphorylated form of eNOS (P-eNOS), a marker of the activated form of eNOS, and eNOS expression. With this method, He and colleagues [25] demonstrated that despite unchanged eNOS expression, the P-eNOS/eNOS ratio was decreased in aortas from mCIA. Somewhat surprisingly, studies that performed the direct measurement of NOS activity or NO production in aortas from AIA rats are lacking. Recently, by using the non-selective competitive NOS inhibitor, L-N_G_-nitroarginine methyl ester (L-NAME), one study provided an indirect argument for a blunted NOS activity in aortas from AIA rats [23]. Alternatively, plasma levels of oxidative degradation products of NO, including nitrite and nitrate (NOx), have been proposed as surrogate markers of vascular NOS activity [29]. However, plasma (nitrite + nitrate) levels were found to be increased (approximately two- to three-fold) at day 22 [30] and day 24 post-injection in AIA rats [20] despite a reduced response to ACh, thus questioning the measurement of plasma NOx levels as a reliable tool to assess ED in arthritis.

#### Uncoupling of endothelial nitric oxide synthase protein and oxidative stress

Under certain conditions, NOS loses its ability to convert L-arginine to L-citrulline but removes an electron from NADPH (reduced form of nicotinamide adenine dinucleotide phosphate) and donates it to molecular oxygen to yield O_2_^–^ instead of NO [31]. This phenomenon is called ‘NOS uncoupling’ and results both in increased O_2_^–^ production and in decreased NO availability. Under these conditions, NO can react with O_2_^–^ to form peroxynitrite (ONOO^−^), which itself is detrimental to the cell and contributes to nitrative stress [32]. As uncoupling of eNOS has been linked to its monomerization after disruption of eNOS dimers, the measurement by Western blotting of the ratio eNOS dimers/eNOS monomers is used as an index of uncoupling [33]. Consistent with eNOS uncoupling in AIA, the aortic ratio of eNOS dimers/monomers was found to be decreased on day 35 post-injection [18]. The most prominent cause of NOS uncoupling is the loss of the critical NOS cofactor BH_4_[34]. In the AIA model, on day 35 post-injection, O_2_^–^ production was measured in homogenates of aortas incubated with or without NOS substrate and various inhibitors [18]. The spontaneous aortic production of O_2_^–^ was enhanced in AIA rats compared with controls. Consistent with a role of NOS uncoupling, incubation with the NOS inhibitor L-NAME reduced O_2_^–^ production. The incubation with BH_4_ also reduced O_2_^–^ production, suggesting a contribution of BH_4_ deficiency to NOS uncoupling. This hypothesis was confirmed by the twofold decrease in serum BH_4_ levels in AIA rats compared with controls [18]. Moreover, treatment of AIA with BH_4_ led to the recovery of normal endothelial function, albeit not modifying the severity of arthritis [18]. The uncoupling of eNOS is not the only possible source of vascular O_2_^–^. NADP (H) oxidase has been reported as one of the most important sources of excess O_2_^–^ production in the vasculature [35]. In AIA, mRNA expression of the aortic NADP (H) oxidase subunits p22phox, gp21phox, and p47phox was increased [19,21], as was the activity of the enzyme [21]. Taken together, these results suggest that both eNOS uncoupling and NADP (H) oxidase overexpression are the predominant sources of O_2_^–^ production in aortas from AIA rats. The role of excessive O_2_^–^ production in ED was confirmed by our demonstration that incubation of aortic rings of AIA rats with Tempol, a membrane-permeable superoxide dismutase mimetic, or with apocynin, an NAPD (H) oxidase inhibitor, significantly improved ACh-induced vasorelaxation [23]. Of note, ONOO^−^ can also directly lead to uncoupling of NOS. The vascular levels of nitrotyrosine, a marker of ONOO^−^ formation, are enhanced in aortas of AIA rats [18]. However, whether ONOO^−^ contributes to ED was not evaluated.

#### Increased arginase pathway

Besides BH_4_ depletion and oxidative stress as causes for eNOS uncoupling, depletion of the NOS substrate L-arginine secondary to arginase upregulation is a cause for NOS uncoupling and ED. Arginase (EC 3.5.3.1) is a hydrolytic enzyme responsible for converting L-arginine to L-ornithine and urea [36]. Mammalian arginases exist in two distinct isoforms (type I and type II) which have specific subcellular localizations and tissue distributions. Notably, both arginase isoforms are expressed by endothelial and vascular smooth muscle cells (VSMCs) [37]. During the last decade, evidence emerged that increased arginase expression/activity contributes to ED associated with various CVDs [37]. Increased aortic arginase activity and increased expression of type II arginase were reported at day 35 post-injection in AIA rats [22]. It is noteworthy that both activity and expression were positively correlated with the intensity of arthritis but not with plasma interleukin-6 levels, suggesting that mechanisms other than systemic inflammation are involved in the upregulation of vascular arginase pathway in AIA. Incubation of aortic rings with N_w_-hydroxy-nor-L-arginine (nor-NOHA), a selective competitive arginase inhibitor, improved ACh-induced vasorelaxation [23]. These data were confirmed in a study in which the treatment of AIA rats with nor-NOHA for 21 days after the onset of arthritis normalized the endothelial function despite the lack of impact of arthritis severity. Consistent with the contributing role of arginase overexpression in NOS uncoupling, increased NOS activity and decreased O_2_^–^ production were identified as mechanisms contributing to the beneficial effect of arginase inhibitor on endothelial function [23].

### The role of endothelium-derived hyperpolarizing factor

Despite the ongoing debate of the molecular identity and signaling pathways, the contribution of EDHFs to the endothelium-dependent relaxation is considered an important feature of normal endothelium function [38]. EDHF has been demonstrated unequivocally in various blood vessels from different species, including humans [39]. The acronym ‘EDHF’ is applied to a factor which induces vascular relaxation in the presence of cyclo-oxygenase (COX) plus NOS inhibitors and which is inhibited by charybdotoxin (an inhibitor of high/intermediate conductance Ca^2+^-activated K^+^ channels) + apamin (an inhibitor of small conductance Ca^2+^-activated K^+^ channels). EDHF induces a potassium-mediated event associated with a reduction in intracellular K^+^ in VSMCs [40]. The role of EDHF in endothelial maintenance has been introduced as a back-up mechanism during NO deficiency [38]. Recent data reported the impairment of aortic EDHF production after ACh challenge in AIA rats, suggesting that the EDHF-mediated compensatory dilator system is lacking in RA [23]. Interestingly, the treatment with an arginase inhibitor restored the EDHF contribution to that of control rats, suggesting a cross-talk between NO and EDHF pathways in arthritis [23].

### The role of prostanoids

In addition to NO and EDHFs, endothelium-derived prostaglandins and TXA_2_ are critical regulators of vascular tone in both physiological and pathological conditions [41]. Physiologically, vasorelaxant prostanoids such as PGI_2_ and vasoconstrictive prostanoids such as TXA_2_ are synthesized by COXs. COX-1 is expressed constitutively and is usually abundant in all animal and human endothelial cells, whereas endothelial COX-2 is induced mainly during inflammatory response [41]. In a model of rabbits with both chronic AIA and atherosclerosis, increased COX-2 expression was reported in the femoral artery [42]. In AIA rats, incubation of aortic rings with the preferential COX-2 inhibitor NS-398 improved ACh-induced vasodilation, thereby indicating that COX-2 overactivation contributes to ED [23]. Likewise, treatment of AIA rats with the COX-2 inhibitor nabumetone from day 15 to 29 post-immunization normalized the vascular response to ACh [16]. By using the TX synthase inhibitor furegrelate, we demonstrated a deleterious role of TX synthase overactivation in endothelial function in AIA [23]. Somewhat surprisingly, overactivation of PGI_2_ synthase is also involved in AIA-associated ED. Indeed, the PGI_2_ synthase inhibitor tranylcypromine improved ACh-induced vasodilation in aortic rings from AIA rats [23]. These results confirmed the janus face of PGI_2_ and suggest that, as already described in animal models of hypertension [43], PGI_2_ can induce vasoconstriction in AIA rats.

### The role of the renin-angiotensin-aldosterone system

The renin-angiotensin-aldosterone system (RAAS) plays an important role in the physiology and pathology of the CV system. ANG-II regulates blood pressure and electrolyte homeostasis and contributes to the inflammatory response in the vascular wall [44]. ANG-II enhances O_2_^–^ production by stimulation of NADP (H) oxidase, thereby causing ED. In ‘traditional’ CVD, treatment with angiotensin-converting enzyme (ACE) inhibitors or angiotensin receptor blockers (ARBs) led to a reduction of CV events [44]. In AIA rats, vascular mRNA expression of ANG-II type 1 receptors and mRNA expression/activity of ACE were increased as compared with controls [21]. In addition, the perfusion of AIA rats with ANG-II for 21 days from the onset of arthritis exacerbated these dysregulations of RAAS while arthritis symptoms were not affected. Moreover, treatment of AIA rats with ARBs led to the improvement of aortic endothelial function along with a decrease in aortic O_2_^–^ production. Collectively, these data suggest a contributing role of ANG-II in AIA-induced ED [21].

## Pharmacological approaches to treat endothelial dysfunction in animal models of arthritis

As shown in Table 2, only a few studies investigated the effects of drugs on AIA-associated ED. Treatment of AIA rats with BH_4_[18], fluvastatin [19], losartan [21], irbesartan [21], or arginase inhibitor [23] administered after the onset of arthritis fully restored the endothelial response to ACh without any influence on the course of arthritis. Recently, the same finding was found after a preventive treatment with simvastatin in mCIA [25]. Such dissociation between vascular and clinical effects of these drugs is very interesting since it suggests that patients with RA, even resistant to anti-rheumatic medications with regard to articular symptoms, could benefit from therapies specifically developed to target ED. Two studies investigated the effects of vitamin E on AIA-associated ED but led to controversial results [15,17]. Given the paramount importance of reducing CV risk in RA, there is a need to determine the effect of anti-rheumatic medications on ED. In patients with RA, studies investigating the impact of disease-modifying anti-rheumatic drugs (DMARDs) or biologic agents on ED are scarce and led to conflicting results [7,45-47]. It is somewhat surprising that studies evaluating the effect of anti-rheumatic drugs on the ED in animal models of arthritis are lacking. Such studies need to be performed.

**Table 2:** Effects of treatments on vascular reactivity in animal model of arthritis

**Authors (year)**	**Animal strain**	**Arthritis model *Arthritogenetic agent *** **( *injection zone *)**	**Treatment**	**Vascular reactivity**	**Arthritis severity**
Cinar *et al*. [15] (1998)	Male rat (strain NR)	AIA *M. tuberculosis* (*pad*)	Vitamin E 100 mg/kg per day (im) from day 0 to day 26 post-injection	↓ ACh, ↑ PE	↓
Ulker *et al*. [16] (2000)	Male Wistar rat	AIA *M. tuberculosis* (*pad*)	Nabumetone 120 and 240 mg/kg per day (po) from day 14 to day 28 post-injection	Normalization of responses to Ach and SNP	= (120 mg/kg per day) ↓ (240 mg/kg per day)
Can *et al*. [17] (2002)	Male Wistar rat	AIA *M. tuberculosis* (*pad*)	Vitamin E 100 mg/kg per day (im) from day 0 to day 26 post-injection	↑ ACh	↓
Nozaki *et al*. [20] (2007)	Male Lewis rat	AIA *M. butyricum* (*tail*)	Keishibukuryogan 1–1.2 g/kg per day (po) from day 0 to day 25 post-injection	↑ ACh	=
Haruna *et al*. [18] (2006)	Male Lewis rat	AIA *M. butyricum* (*tail*)	BH_4_ 20 mg/kg (ip) from day 21 to day 35 post-injection	↑ ACh = SNP	=
Haruna *et al*. [19] (2007)	Male Lewis rat	AIA *M. butyricum* (*tail*)	Fluvastatin 5 mg/kg per day (po) from day 21 to day 35 post-injection	↑ ACh	=
Sakuta *et al*. [21] (2010)	Male Lewis rat	AIA *M. butyricum (tail*)	Losartan 3 mg/kg per day (po) and Irbesartan 5 mg/kg per day (po) from day 14 to day 35 post-injection	↑ ACh = SNP	=
Prati *et al*. [23] (2012)	Male Lewis rat	AIA *M. butyricum* (*tail*)	Nor-NOHA 40 mg/kg per day (ip) from day 14 to day 35 post-injection	↑ ACh = SNP = NE, ANG-II, ET-1	=
He *et al*. [25] (2013)	Male DBA/1 mice	CIA *Bovine collagen (tail)*	Simvastatin 50 mg/kg per day (po) from day 7 before injection to day 56 post-injection	↑ ACh = SNP	=

Vascular reactivity was studied in the model of isolated aortic rings. ↑, increased; ↓, decreased; =, unchanged; ACh, acetylcholine; AIA, adjuvant-induced arthritis; ANG-II, angiotensin II; BH_4_, tetrahydrobiopterin; CIA, collagen-induced arthritis; ET-1, endothelin-1; im, intramuscular; ip, intraperitoneal; KCl, high potassium chloride; NE, norepinephrine; nor-NOHA, Nw-hydroxy-nor-L-arginine; NR, not reported; PE, phenylephrine; po, *per os*; SNP, sodium nitroprusside.

## Conclusions

The studies presented in the present review provide compelling evidence that aortic ED is impaired during the severe inflammatory phase of the AIA model, in agreement with the presence of a macrovascular ED in patients with established RA [7]. The available data obtained in animal models identified several mechanisms of ED: decreased endothelial NO production, excessive endothelial O_2_^–^ production, deficiency in the NOS cofactor BH_4_, upregulation of arginase, upregulation of NADP (H) oxidase, and overactivation of COX-2, TX synthase, and PGI_2_ synthase, thereby identifying future potential targets for new therapeutic options to treat ED in patients with arthritis. However, many points are still unresolved and would benefit from further studies on animal models of RA. A first point concerns the time course of ED with respect to the arthritis symptoms and diagnosis. Is ED occurring early or even before the onset of the disease? Is it long-lasting and persistent even though the inflammatory phase is resolved? A second point concerns the presence or not of ED in the microvasculature. Microvascular dysfunction plays an important role in the development of target organ damage in the heart and kidney as well as in the development of CV risk factors [48,49]. Whether microvascular dysfunction occurs early in the course of RA is not known and whether microvascular ED mirrors macrovascular ED, and as such occurs at the same time in the disease process, remain to be determined. A third point concerns the link between inflammatory process and the occurrence of ED. Animal studies revealed that the improvement of endothelial function occurs even though the severity of arthritis is unchanged, suggesting that the reduction of inflammation is not a prerequisite for the improvement of endothelial function. Moreover, the role of the different actors of atherogenesis such as cellular adhesion molecules, cytokines, chemokines, vascular endothelial growth factor, autoantibodies, and different cells of the immune response such as macrophages or dendritic cells [46] in ED has to be studied in animals. Finally, animal models may help to define the effect of immunosuppressive drugs such as DMARDs, anti-tumor necrosis factor-alpha, or glucocorticoids on ED and the mechanisms behind their effects.

## Abbreviations

ACE: Angiotensin-converting enzyme; ACh: Acetylcholine; AIA: Adjuvant-induced arthritis; ANG-II: Angiotensin II; ARB: Angiotensin receptor blocker; BH4: Tetrahydrobiopterin; COX: Cyclo-oxygenase; CV: Cardiovascular; CVD: Cardiovascular disease; DMARD: Disease-modifying anti-rheumatic drug; ED: Endothelial dysfunction; EDHF: Endothelium-derived hyperpolarizing factor; eNOS: Endothelial nitric oxide synthase; iNOS: Inducible nitric oxide synthase; L-NAME: L-N_G_-nitroarginine methyl ester; mCIA: Collagen-induced arthritis in mice; NO: Nitric oxide; nor-NOHA: N_w_-hydroxy-nor-L-arginine; NOS: Nitric oxide synthase; NOx: Nitrite plus nitrate; O2–: Superoxide anion; ONOO−: Peroxynitrite; P-eNOS: Serine 1177-phosphorylated form of endothelial nitric oxide synthase; PGI2: Prostacyclin; RA: Rheumatoid arthritis; RAAS: Renin-angiotensin-aldosterone system; TXA2: Thromboxane A_2_; VSMC: Vascular smooth muscle cell.

## Competing interests

The authors declare that they have no competing interests.

## Authors’ contributions

PT and CD designed the review and drafted the manuscript. KM-G, CP, and DW participated in designing the review and helped with drafting the manuscript. All authors read and approved the final manuscript.
